# Knockdown of Adenosine 5′-Triphosphate-Dependent Caseinolytic Protease Proteolytic Subunit 6 Enhances Aluminum Tolerance in Peanut Plants (*Arachis hypogea* L.)

**DOI:** 10.3390/ijms251910416

**Published:** 2024-09-27

**Authors:** Yusun Shi, Dayue Zhang, Ronghua Liang, Dong Xiao, Aiqin Wang, Longfei He, Jie Zhan

**Affiliations:** 1 College of Agriculture, Guangxi University, Nanning 530004, China; shiyusun2012@gmail.com (Y.S.); 13880122570@163.com (D.Z.); m15678469972@163.com (R.L.); xiaodong@gxu.edu.cn (D.X.); waiqing1966@126.com (A.W.); 2 Guangxi Key Laboratory of Agro-Environment and Agro-Products Safety, Guangxi University, Nanning 530004, China; 3 Key Laboratory of Crop Cultivation and Tillage, Guangxi University, Nanning 530004, China

**Keywords:** Al^3+^ toxicity response, AhClpP6, photosynthesis, ultrastructure

## Abstract

Aluminum (Al^3+^) toxicity in acidic soils reduces root growth and can lead to a considerable reduction in peanut plants (*Arachis hypogea* L.). The caseinolytic protease (Clp) system plays the key role in abiotic stress response. However, it is still unknown whether it is involved in peanut response to Al^3+^ stress. The results from this study showed that Adenosine 5′-triphosphate (ATP)-dependent caseinolytic protease proteolytic subunit 6 (AhClpP6) in peanut plants was involved in the Al^3^ stress response through its effects on leaf photosynthesis. The *AhClpP6* expression levels in the leaf and stem significantly increased with the Al^3+^ treatment times. Knockdown *AhClpP6* peanut lines accumulated significantly more Al^3+^ when exposed to Al^3+^ stress, which reduced leaf photosynthesis. Furthermore, in response to Al^3+^ treatment, knockdown of *AhClpP6* resulted in a flattened shape of chloroplasts, disordered and flattened thylakoid, and accumulating more starch grains than those of the wild-type (WT) peanut lines. Taken together, our results suggest that *AhClpP6* regulates Al^3+^ tolerance by maintaining chloroplast integrity and enhancing photosynthesis.

## 1. Introduction 

Aluminum (Al) in acidic soils reduces agricultural production across the globe [1]. Defensive measures taken by plants against Al^3+^ toxicity include the exudation of organic acid by roots to chelate soil Al ions [2]. Toxic Al^3+^ is released when the soil pH < 5 and can inhibit root elongation [3,4]. The main site affected by Al^3+^ toxicity is the root-apex transition zone (TZ), and the growth further seriously limits the nutrient and water uptake of roots [5]. The rice (*Oryza sativa* L.) seedling root system is sensitive to changes in Al concentration, and this is manifested as obvious root growth inhibition, shortening of the roots, deepening of the root color, abnormal root tips, and root-crown detachment [6]. Al^3+^ treatment on the soybean (*Glycine max* L.) significantly inhibited root elongation, with an increase in larger plasma membrane permeability [7].

Al^3+^ toxicity also affects the aboveground parts of plants. Under acidic conditions, Al^3+^ toxicity led to increased leaf temperature, chlorophyll degradation, and limited CO_2_ assimilation [8]. Al^3+^ stress decreased the net photosynthesis rates and increased the intercellular CO_2_ concentration in Al-sensitive alfalfa (*Medicago sativa* L.) [9]. A large amount of Al^3+^ (7.31 mg/g) could be transferred from the root to the aged leaves with 1.5 mg/L Al^3+^, which resulted in dissolving the leaf thylakoid lamellae and disintegrating the chloroplasts [10]. Pereira et al. [11] found that the Al^3+^-induced inhibition of photosynthesis caused damage to the thylakoid structure, which affected the photosynthetic transport of electrons in *Citrus* leaves.

The caseinolytic protease (Clp) system is crucial for plant growth and development, likely facilitating the degradation of numerous chloroplast proteins, including misfolded proteins, protein aggregates, and potentially smaller proteins and peptides [12]. The Clp system is involved in chloroplast homeostasis [13] during the degradation of partially accumulated complexes and damaged proteins [14,15]. Halperin et al. [16] found that each subunit has a unique function in the Clp complex in pea (*Pisum sativum*) chloroplasts. *ClpC1* mutants exhibited significant defects in chloroplast proteostasis, resulting in markedly reduced growth and pigment levels and increased accumulation of chloroplast and cytosolic chaperones. *ClpC2* mutants were susceptible to inhibition of chloroplast translation by lincomycin [17].

Clp also plays an important role in stress. Suppressing the expression of *ClpB3* in *Chlamydomonas reinhardtii* could decrease accumulation of the ribosomal subunit PRPL1 and increase the stromal protease DEG1C during heat stress [18]. Overexpression of *ZnJClpB1*-*C* in tobacco (*Nicotiana tabacum*) exhibited a significantly higher chlorophyll content, thereby significantly improving tolerance to heat stress [19]. Knockout of *OsClpP6* in rice (*Oryza sativa* L.) increased the resistance of rice to brown planthoppers by enhancing the level of brown-planthopper-induced jasmonic acid, jasmonic acid-isoleucine, and abscisic acid [20]. However, there have been relatively few studies on the roles played by Clp in response to Al^3+^ stress.

The peanut (*Arachis hypogea* L.) is a globally important economic crop due to its high oil (45–55%) and protein content (20–35%) [21]. However, in recent decades, peanut yields have decreased by about 15% as a result of soil acidification of croplands of South China [22]. Here, to explore this issue, we cloned the peanut gene *AhClpP6*, which encodes the ATP-dependent caseinolytic protease proteolytic subunit 6, and investigated its role in the tolerance of peanut plants to Al^3+^ toxicity. The hypothesis was that AhClpP6 mediated, at least in part, chloroplast integrity under Al^3+^ stress, thereby changing photosynthetic efficiency.

## 2. Results

### 2.1. Phylogenetic Analysis of AhClpP6

**Figure 1:**
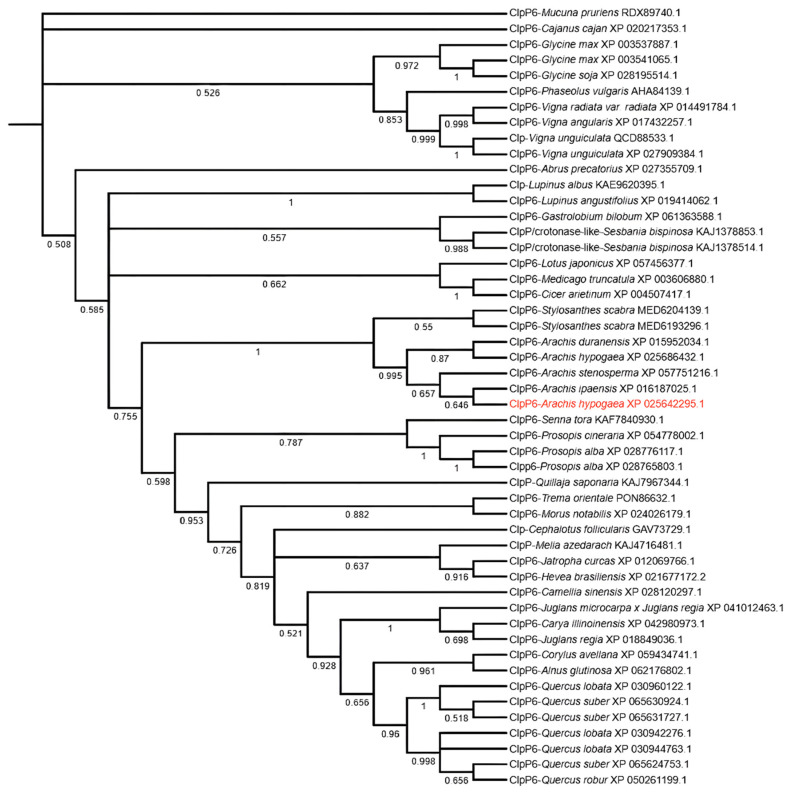
Phylogenetic analyses of the ClpP6 family proteins from peanut plants and other plant species. Red font letters mean AhClpP6 of *Arachis ipaensis* ClpP6.

Data obtained from the phylogenetic analysis of AhClpP6 using MEGA 7.0 software revealed that peanut (*Arachis hypogea* L.) AhClpP6 is most similar to that of *Arachis ipaensis* ClpP6 (XP_016187025.1) and *Arachis stenosperma* ClpP6 (XP_057751216.1) (Figure 1). In contrast, AhClpP6 had the lowest phylogenetic relationship with *Vigna radiata* var. radiata ClpP6 (XP_014491784.1), *Vigna angularisj* ClpP6 (XP_017432257.1), *Vigna angularisj* ClpP6 (XP_052734245.1), *Vigna unguiculata* ClpP6 (XP_027909384.1), and *Vigna unguiculata* Clp (QCD88533.1). The family relationships with *Glycine soja* Clpp6 (XP_028195514.1), *Glycine max* Clpp6 (XP_003541065.1), *Glycine max* ClpP6 (XP_003537887.1), and *Cajanus cajan* ClpP6 (XP_020217353.1) were also relatively distant. The relationship between *Arachis duranensis* ClpP6 (XP_015952034.1) and AhClpP6 was closer than that between *Stylosanthes scabra* ClpP6 (MED6193296.1) and *Stylosanthes scabra* ClpP6 (MED6204139.1).

### 2.2. AhClpP6 Expression Increased with Al^3+^ Stress in Peanut Plants

**Figure 2:**
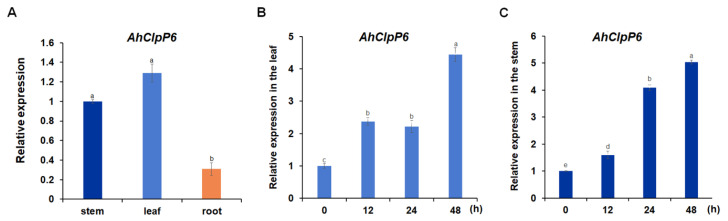
*AhClpP6* expression profile in peanut plants. (**A**) in root, stem, and leaf tissues. (**B**,**C**) in the leaves (**B**) and stems (**C**) under 100 μM AlCl_3_ treatment for 0, 12, 24, and 48 h. Values show means ± SD (*n* = 3). Different letters indicate significant differences at *p* < 0.05. All experiments were repeated at least three times with similar results.

As shown in Figure 2A, the highest expression levels of *AhClpP6* were in peanut leaves and stems. The *AhClpP6* expression levels in the leaves and stems significantly increased with Al^3+^ treatment time in the wild type (WT, Figure 2B,C). Furthermore, the highest expression levels, which were 4.43 and 5.03, respectively, occurred at 48 h after treatment, suggesting that *AhClpP6* is modulated during the Al^3+^ stress response.

### 2.3. Sequencing and Identification of Differentially Expressed Genes

**Figure 3:**
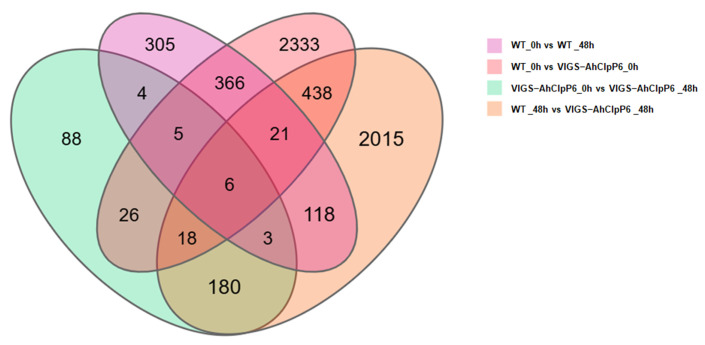
Venn diagram showing the relationships between different groups of DEGs. WT, the wild-type peanut; VIGS-AhClpP6, knockdown of *AhClpP6* in peanut seedlings; 0 h, without Al^3+^ stress; 48 h, under 100 μM AlCl_3_ treatment for 48 h.

The AhClpP6 sequence and RNA-seq data were analyzed to elucidate the biological functions of AhClpP6 in plant growth and development. Expression profiling was conducted using RNA-seq datasets for leaves from the WT and VIGS-AhClpP6 lines. The overlapping DEGs between the differential analysis groups are shown in a Venn diagram (Figure 3). The DEGs across different cultivars and time points were compared, and four groups of DEGs were identified, with the VIGS-AhClpP6_0h vs. WT_0h group having the largest number of DEGs. A Venn diagram was used to determine the overlapping DEGs across the four differential analysis groups. A comparison of the WT and VIGS-AhClpP6 lines at different time points under Al^3+^ stress showed that there were 5926 DEGs across the four groups, with 6 DEGs common to all four groups. These findings indicate that gene silencing of *AhClpP6* has a more significant impact on DEGs than Al^3+^ stress alone.

### 2.4. GO and KEGG Annotation of DEGs

**Figure 4:**
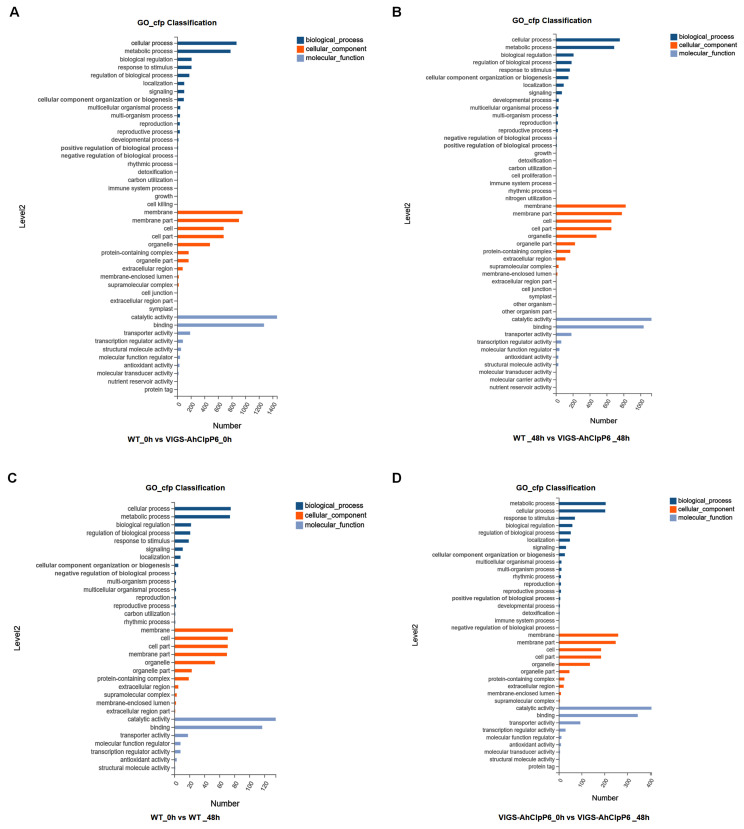
GO enrichment of DEGs between WT and VIGS-AhClpP6 in response to Al^3+^ stress. (**A**) Enriched GO terms for WT_0h vs. VIGS-AhClpP6_0h; (**B**) WT_48h vs. VIGS-AhClpP6_48h; (**C**) WT_0h vs. WT_48h; and (**D**) VIGS-AhClpP6_0h vs. VIGS-AhClpP6_48h. The *X*-axis represents the number of genes, and the *Y*-axis represents the functional category.

A GO function annotation and enrichment analysis was conducted to further explore the functions of the DEGs. The GO terms with a *p*-value ≤ 0.05 were considered to have been significantly enriched. The GO analysis (Figure 4) revealed that the DEGs encompassed a broad spectrum of biological processes, cellular components, and molecular functions, which were classified into 15, 11, and 7 biological processes, cellular components, and molecular functions categories in the WT_48h vs. WT_0h group, respectively; 18, 10, and 9 categories in the VIGS-AhClpP6_48h vs. VIGS-AhClpP6_0h group; 21, 13, and 10 categories in the WT_0h vs. VIGS-AhClpP6_0h group; and 22, 15, and 10 categories in the WT_48h vs. VIGS-AhClpP6_48h group, respectively. Among biological processes, the largest categories were cellular processes and metabolic processes, while catalytic activity and binding were the predominant categories in molecular functions across all four groups. Cellular component genes were predominantly associated with the membrane and cell in the WT_48h vs. WT_0h group, and with the membrane and membrane parts in the remaining three groups.

**Figure 5:**
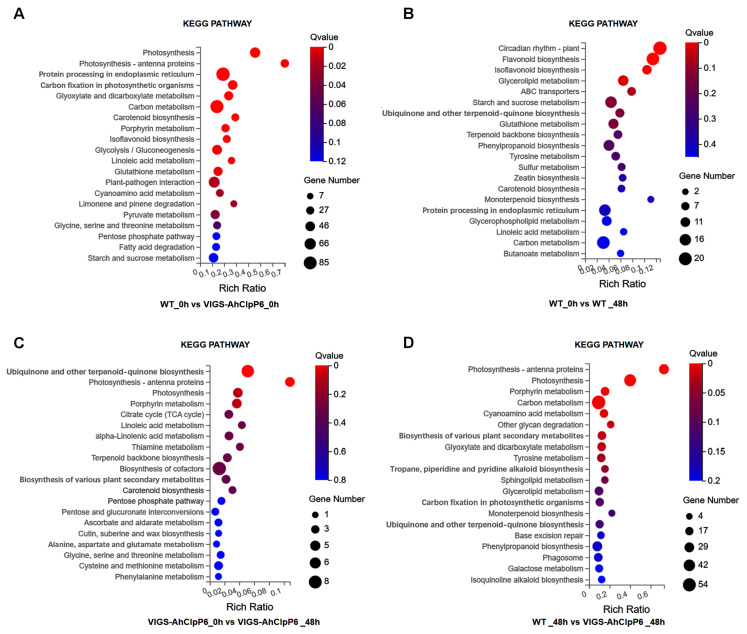
KEGG pathway enrichment of DEGs between WT and VIGS-AhClpP6 seedlings in response to Al^3+^ stress. (**A**) Enriched GO terms for WT_0h vs. VIGS-AhClpP6_0h; (**B**) WT_0h vs. WT_48h; (**C**) VIGS-AhClpP6_0h vs. VIGS-AhClpP6_48h; and (**D**) WT_48h vs. VIGS-AhClpP6_48h. The *X*-axis represents the rich ratio for the genes, and the *Y*-axis represents the functional category.

A KEGG pathway analysis was also performed to assess the biological roles of the DEGs (Figure 5). The top three pathways in the VIGS-AhClpP6_48h vs. VIGS-AhClpP6_0h group were circadian rhythm-plant, flavonoid biosynthesis, and isoflavonoid biosynthesis; the top three pathways for the WT_0h vs. VIGS-AhClpP6_0h group were photosynthesis, photosynthesis-antenna proteins, and protein processing in the endoplasmic reticulum; the top pathways in the WT_48h vs. WT_0h group were ubiquinone and other terpenoid-quinone biosynthesis, photosynthesis-antenna proteins, and photosynthesis; and the top pathways in the WT_48h vs. VIGS-AhClpP6_48h group were photosynthesis-antenna proteins, photosynthesis, and porphyrin metabolism. A total of 232 DEGs related to photosynthesis and chloroplast development were identified. Of these, 222 DEGs were concurrently detected in both the WT_0h vs. VIGS-AhClpP6_0h and WT_48h vs. VIGS-AhClpP6_48h groups, with 59 DEGs showing significant expression changes in both groups. Collectively, these results suggest that Al^3+^ stress significantly affects leaf photosynthesis and chloroplast development in *AhClpP6* knockdown seedlings.

**Figure 6:**
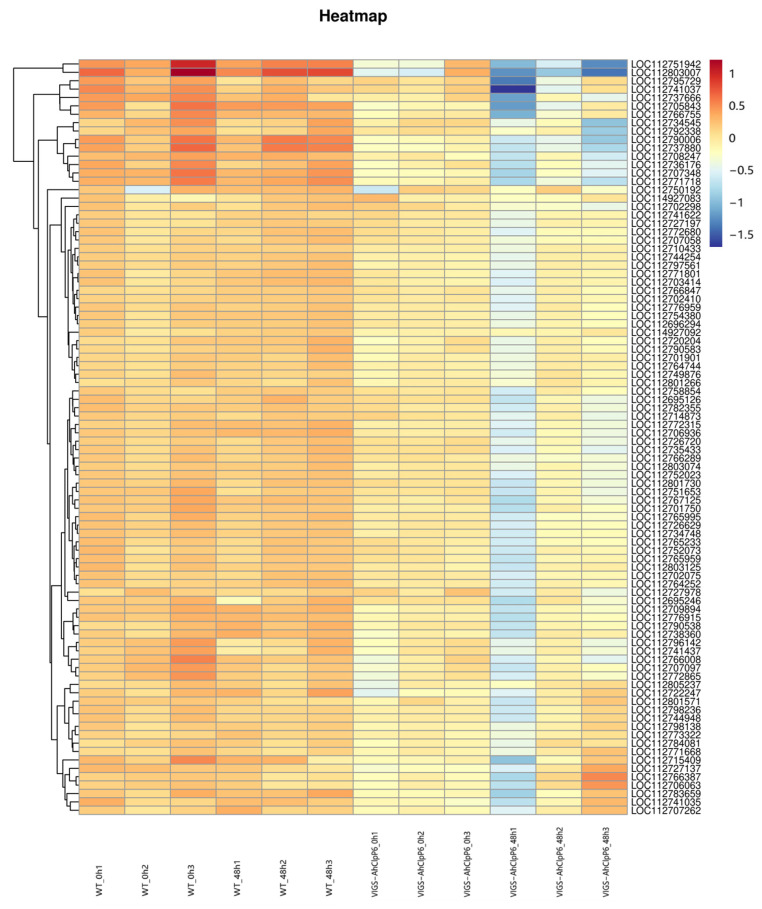
Heatmap indicating transcriptional levels of WTandVIGS-AhClpP6 seedlings in response to Al^3+^ stress. The heatmap shows the log10 fold change of each gene.

Based on previous results of KEGG pathway enrichment, 90 genes whose pathway is photosynthesis and photosynthesis-antenna proteins were selected (Figure 6). Among the 90 DEGs, there were 36 upregulated genes (0.002 ≤ log2fold-change ≤ 0.535) and 54 downregulated genes (−0.88 ≤ log2fold-change ≤ −0.0007) in WT_0h compared with WT_48h; 90 upregulated genes (0.05 ≤ log2fold-change ≤ 3.43) in VIGS-AhClpP6_0h compared with WT_0h; 16 upregulated genes (0.03 ≤ log2fold-change ≤ 1.13) and 74 downregulated genes (−3.87 ≤ log2fold-change ≤ −0.03) in VIGS-AhClpP6_0h compared with VIGS-AhClpP6_48h; 88 upregulated genes (0.27 ≤ log2fold-change ≤ 6.57) and 2 downregulated genes (−0.22 ≤ log2fold-change ≤ −0.13) in VIGS-AhClpP6_48h compared with WT_48h. In particular, two chlorophyll a-b binding protein of LHCII type 1 genes (LOC112803007 and LOC112751942) were most significantly upregulated both in VIGS-AhClpP6_0h vs. WT_0h, with log2fold-change ranging from 2.73 to 3.43, and VIGS-AhClpP6_48h compared with WT_48h, with log2fold-change ranging from 4.81 to 6.57. But these two genes were not significantly downregulated in WT_0h compared with WT_48h, with log2fold-change ranging from −0.78 to −0.72, while being significantly downregulated in VIGS-AhClpP6_0h compared with VIGS-AhClpP6_48h, with log2fold-change ranging from −3.87 to −2.85.

### 2.5. AhClpP6 Positively Regulates Al^3+^ Tolerance by Modulating Photosynthesis

**Figure 7:**
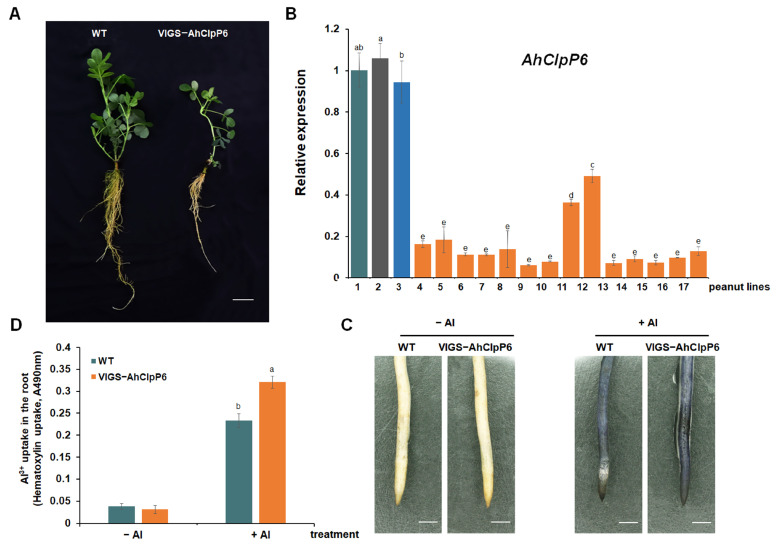
Functional characterization of *AhClpP6* in peanut plants. (**A**) Phenotypes of knockdown of *AhClpP6* peanut lines (VIGS-AhClpP6); scale bar, 5 cm. (**B**) RT-qPCR analysis of *AhClpP6* expression in peanut leaves; *X*-axis represents different peanut lines. Peanut lines: 1, WT; 2, empty; 3, positive control; 4–17, VIGS-AhClpP6. (**C**,**D**) Effects of Al^3+^ stress on plant growth. (**C**) Hematoxylin staining of the root tips; bar, 1 mm. (**D**) Al^3+^ uptake in the root tips. −Al means without Al^3+^ treatment; +Al means under 100 μM AlCl_3_ treatment for 48 h. Values are means ± SD (*n* = 3). Different letters indicate significant differences at *p* < 0.05. All experiments were repeated at least three times with similar results.

Knockdown *AhClpP6* lines (VIGS-AhClpP6 lines) were produced to investigate the function of AhClpP6 in peanut plants (Figure 7A). *AhClpP6* transcription was significantly lower in the leaves from most of the pTRV:AhClpP6 seedling lines compared to the WT plants (Figure 7B). When exposed to Al^3+^ stress, the hematoxylin staining intensity was clearly stronger, and the root tips accumulated significantly more Al^3+^ in the *AhClpP6* knockdown seedlings than in the WT seedlings (Figure 7C). Furthermore, VIGS-AhClpP6 root tips accumulated significantly (1.3-fold) more Al^3+^ than the WT seedlings when they were exposed to Al^3+^ (Figure 7D). These results demonstrate that *AhClpP6* positively affects peanut tolerance to Al^3+^ stress.

**Figure 8:**
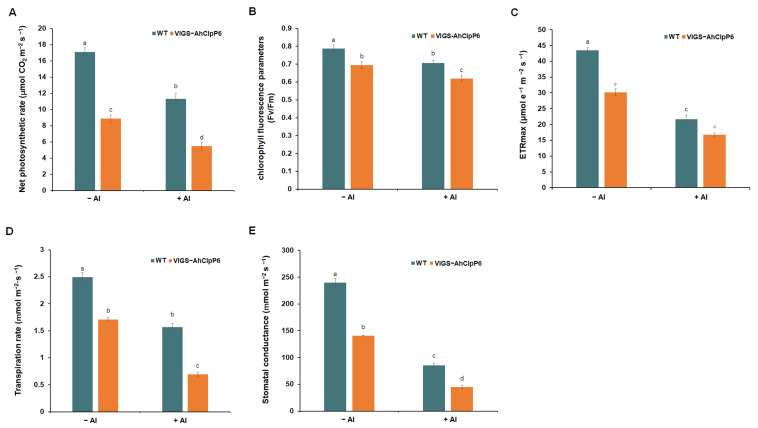
Photosynthesis detection in the peanut *AhClpP6* knockdown line under 100 μM AlCl_3_ treatment for 48 h. (**A**) Net photosynthetic rate; (**B**) chlorophyll fluorescence parameters; (**C**) maximum electron transport rate (ETRmax); (**D**) transpiration rate; (**E**) stomatal conductance. −Al means without Al^3+^ treatment; +Al means under 100 μM AlCl_3_ treatment for 48 h. Values are means ± SD (*n* = 3). Different letters indicate significant differences at *p* < 0.05. All experiments were repeated at least three times with similar results.

Photosynthesis decreased after 24 h of Al^3+^ treatment in the WT seedlings, but this decrease was significantly greater in the *AhClpP6* knockdown lines (VIGS-AhClpP6) (Figure 8). The net photosynthetic rate, the Fv/Fm value, and the ETR_max_ for the VIGS-AhClpP6 lines were significantly lower than those for the WT, which was consistent with their chlorotic appearance. The net photosynthetic rates for both the WT and VIGS-AhClpP6 lines were also significantly lower after Al^3+^ treatment. The PSII photochemical efficiency and the ETR_max_ values for the leaves from the WT and VIGS-AhClpP6 lines were also significantly inhibited after Al^3+^ stress. Furthermore, compared to the control plants, Al^3+^ treatment decreased the transpiration rate and stomatal conductance by 44% and 53% in the *AhClpP6* mutant lines, respectively. In summary, these results suggest that *AhClpP6* positively affects photosynthesis. The above results show that AhClpP6 enhances peanut Al^3+^ tolerance by upregulating photosynthesis.

### 2.6. AhClpP6 Positively Regulates Chloroplast Integrity

**Figure 9:**
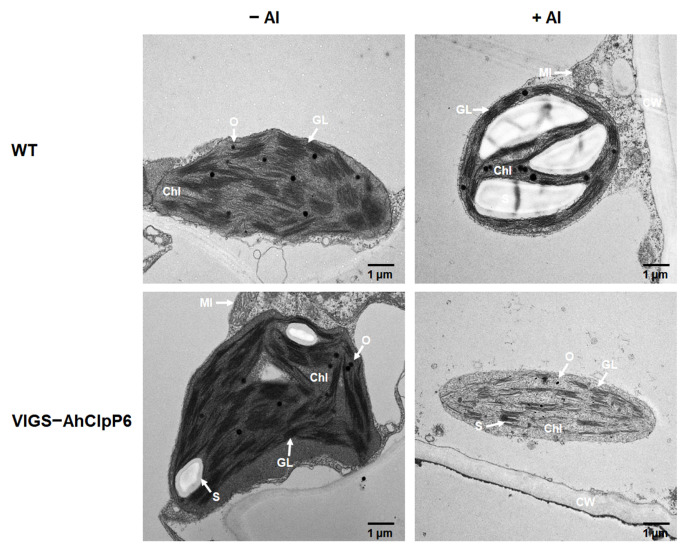
Microscopic analysis of the effect of the *AhClpP6* knockdown line on Al^3+^-induced chloroplast structure changes in leaves. −Al means without Al^3+^ treatment; +Al means under 100 μM AlCl_3_ treatment for 48 h. Bar, 1 μm. Values are the mean ± SD (*n* = 3). Different letters indicate significant differences at *p* < 0.05. All experiments were repeated at least three times with similar results. Chl, chloroplast; GL, grana lamellae; O, osmiophilic granule; S, starch grain.

**Figure 10:**
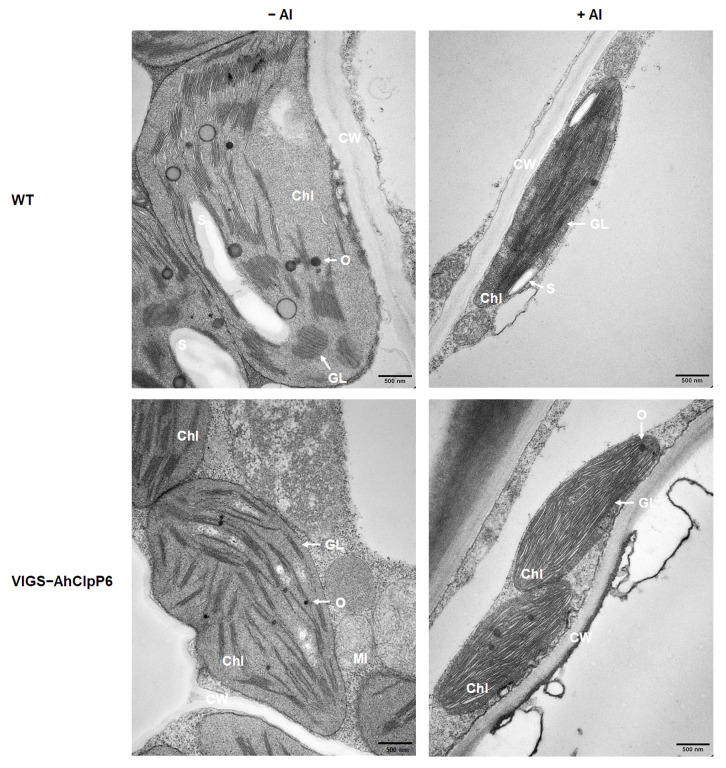
Microscopic analysis of the effect of the *AhClpP6* knockdown line on Al^3+^-induced chloroplast structure changes in stems. −Al means without Al^3+^ treatment; +Al means under 100 μM AlCl_3_ treatment for 48 h. Bar, 500 nm. Values are the mean ± SD (*n* = 3). Different letters indicate significant differences at *p* < 0.05. All experiments were repeated at least three times with similar results. Chl, chloroplast; GL, grana lamellae; O, osmiophilic granule; S, starch grain.

The chloroplast ultrastructure in the leaves and stems was observed (Figure 9 and Figure 10). The chloroplasts in the WT leaves and stems had the expected morphology and a well-structured thylakoid, but the chloroplasts in VIGS-AhClpP6 seedlings showed dramatic changes, with flattened and disordered chloroplasts (Figure 9 and Figure 10). Moreover, chloroplasts in the +Al treatment accumulated more starch grains than those in the −Al treatment in leaves of VIGS-AhClpP6 lines than those of WT (Figure 9). In the Al^3+^ stress stage, the lamellar system accommodated to the flattened shape of the chloroplasts. Taken together, the results indicate that *AhClpP6* positively affects peanut tolerance to Al^3+^ stress by maintaining chloroplast integrity.

## 3. Discussion

The mechanisms of Al^3+^ toxicity from acidic soils are important issues in crop yield and productivity. Recent studies have revealed that chloroplast proteases play an important role in plant adaptation to abiotic stresses by remodeling the chloroplast proteome [23]. In this study, we found that *AhClpP6* positively regulates Al^3+^ tolerance by maintaining chloroplast integrity and controlling photosynthesis, which provided novel insights into the molecular mechanism of plant tolerance to Al^3+^ stress.

*AhClpP6* expression at the transcriptional level in peanut stems and leaves increased under Al^3+^ treatment, with significant upregulation observed at 48 h (Figure 2). This may be due to the time required for Al ions to be transported from the root tips to the leaves and stems. VIGS-AhClpP6 peanut lines displayed inhibited growth under normal conditions, indicating that AhClpP6 protease is vital for plant growth. A similar result was obtained from *Arabidopsis thaliana*. Chloroplasts in the inner leaves of *ClpP6* antisense lines had poorly developed thylakoid membrane networks and reduced overall membrane contents [24]. *AhClpP6* displayed a positive effect on Al^3+^ tolerance in peanut plants (Figure 7), suggesting it was essential for the development of this early Al^3+^ response. Additionally, the annotated gene function analysis showed that 232 DEGs were associated with chloroplast development and photosynthesis. These findings suggest that the positive effects of *AhClpP6* on peanut plants exposed to Al^3+^ stress may be related to photosynthesis and chloroplast growth.

A previous study has also found that Al^3+^ absorption by chloroplasts induces the expression of certain genes in the chloroplast genome, including those related to membrane-bound ATP-independent proteases, which play a critical role in tolerance mechanisms [25]. In this study, under normal conditions, the net photosynthetic rate, Fv/Fm, and ETR values for the WT seedlings were higher than those for the VIGS-AhClpP6 seedlings, indicating that *AhClpP6* knockdown negatively impacts photosynthesis in peanut leaves. Under Al^3+^ stress, the reductions in net photosynthetic rate, Fv/Fm, and ETR_max_ were significantly greater in the VIGS-ClpP6 plants than in the WT plants, which may lead to a decrease in the accumulation of organic substances in peanuts. In addition, the decrease in Fv/Fm indicates a decline in PSII efficiency, and the reduction in ETR_max_ reflects a drop in the maximum photosynthetic rate. These characteristics suggest that AhClpP6 positively affects photosynthesis in peanut leaves and that this impact is further enhanced under Al^3+^ stress. In a similar study, using high Al^3+^ conditions limited CO_2_ assimilation and thus decreased photosynthesis [8]. Interestingly, evidence suggested that drought tolerance in maize (*Zea mays* L.) potentially resulted in Al^3+^ tolerance due to the changes in leaf growth and metabolic and physiological adjustments [26]. One of the most important physiological responses to drought stress is photosynthesis inhibition [27,28].

Based on these results, we hypothesized that AhClpP6 could be required in chloroplast homeostasis under Al^3+^ stress. Therefore, the chloroplast ultrastructure of leaves and stems from the *AhClpP6* knockdown peanut lines was further observed. The chloroplasts in the VIGS-AhClpP6 peanut seedlings were smaller and more flattened and disordered than those in the WT with or without Al treatment (Figure 9 and Figure 10). Taken together, it is proposed that the integrity and order of chloroplasts may have been related to the better performance of peanut plants under Al^3+^ stress, which represents a high potential in coordinating with photosynthesis and therefore enhances plant tolerance under Al^3+^ exposure, and AhClpP6 appears to play an indispensable role in this process. AhClpP6 may be utilized in generating genetically engineered crops with enhanced resistance to Al^3+^ stress.

## 4. Materials and Methods

### 4.1. Peanut (Arachis hypogea L.) Cultivation and Al^3+^ Treatment

Seeds from the peanut (*Arachis hypogea* L.) cultivar ‘Zhonghua-12’ were incubated in wet perlite under dark conditions at 26 ± 2 °C for approximately 3–4 days. The sprouted seedlings were transferred to Hoagland’s nutrient solution under a 12 h/12 h light/dark cycle with a photon flux density of 30–50 µmol/m^2^/s supplied by daylight fluorescent tubes (Philips Chinese Ltd., Shanghai, China) at 26 ± 2 °C. The Hoagland’s nutrient solution was replaced with fresh nutrient solution at daily intervals until the third true leaf appeared. Then, the seedlings were preconditioned for 1 d in 0.5 mM CaCl_2_ solution (pH 4.2–4.5) for 24 h and transferred to a 0.5 mM CaCl_2_ solution that contained 100 μM AlCl_3_ for 0, 12, 24, or 48 h. Each treatment was repeated three times. Approximately 1 cm long root tips were removed and stored at −80 °C for further analysis.

### 4.2. Multiple Sequence Alignments and Phylogenetic Analysis

The AhClpP6 members used for the phylogenetic analysis were obtained from the GenBank (https://www.ncbi.nlm.nih.gov/genbank/, accessed on 10 August 2024) and TAIR (http://www.arabidopsis.org/, accessed on 10 August 2024) databases and were from *Arabidopsis thaliana*. The phylogenetic tree was constructed using MEGA7.0 software and the iTOL v6 online tool (https://itol.embl.de/, accessed on 15 August 2024).

### 4.3. RT-qPCR Analysis

Total RNA was extracted using an Eastep^®^ Super RNA Kit (Promega Biological Products Co., Ltd., Shanghai, China) according to the manufacturer’s instructions. HiScript^®^ II qRT SuperMix (Vazyme Biotech Co., Ltd., Nanjing, China), with RNA (500 ng) as a template, was used to synthesize the cDNA according to the manufacturer’s instructions. An RT-qPCR analysis was performed according to [29], and all primers for PCR amplification were designed using Primer 5.0 software (Table S1). The ubiquitin gene, *AhUBQR10* (accession No. EG030441), was used as an internal control to quantify the relative transcript levels in peanut plants [30]. The relative expression of each gene was calculated using the 2^−ΔΔCt^ method based on data reported by Livak and Schmittgen [29], who used three biological replicates collected from three independent plants.

### 4.4. Virus-Induced Gene Silencing (VIGS)

Peanut plants with virus-induced gene silencing (VIGS) were produced according to Liao et al. [31]. Briefly, *AhClpP6* was amplified from peanut cDNA and ligated into the pTRV2 vector. Then, pTRV1 and the recombinant pTRV2 vectors were transformed into competent cells of *Agrobacterium tumefaciens* strain GV3101. The positive control was *AhPDS* (GenBank accession ON513417, peanut phytoene desaturase gene). The peanut seeds were surface-sterilized with 75% (*v*/*v*) ethanol for 30 s and 0.1% (*v*/*v*) HgCl_2_ for 5–8 min. They were then washed 4–5 times with sterile water, and their outer seed coats were removed. The naked seeds were scratched with a scalpel and then immersed in inoculation solution (OD_600_ of 0.8) for 2 h before transfer to Murashige and Skoog solid medium for 2–3 d in darkness at 22–24 °C. The germinated seeds were suspended in Hoagland’s nutrient solution and grown in a growth chamber at 26 ± 2 °C under a 12/12 light/dark cycle.

### 4.5. RNA-Seq and Transcriptome Analysis

RNA-seq was used to determine the transcriptome data for the VIGS-AhClpP6 peanut plants and WT peanut to further investigate the *AhClpP6* response to Al^3+^ toxicity. The treatment conditions were 100 μM AlCl_3_ for 48 h. There were four treatments consisting of two culture materials and two treatment conditions. These were Al^3+^ (WT, normal culture), A2 (WT, Al^3+^ treatment), B1 (VIGS-AhClpP6 peanut, normal culture), and B2 (VIGS-AhClpP6 peanut, Al^3+^ treatment), and each group was replicated three times (e.g., A1-1, A1-2, and A1-3).

The differentially expressed genes were analyzed using Gene Ontology (GO) and Kyoto Encyclopedia of Genes and Genomes (KEGG) enrichment by the clusterProfiler R package version 2.26.0 (http://github.com/jdstorey/qvalue, accessed on 11 September 2024). Then, TB-tools software v0.665 was used to generate a diagram showing the enriched GO terms. In the Gene Ontology (GO) enrichment analysis, all DEGs were mapped to the GO database (http://www.geneontology.org/, accessed on 20 July 2024), and the number of genes for each GO term was calculated. Then, the hypergeometric test, with a *p*-value of 0.05 as the criterion, was applied to find the significantly enriched GO terms. A KEGG pathway analysis was performed (https://www.kegg.jp/, accessed on 20 July 2024), with a significance level of *p* < 0.05. A heatmap was created using the OmicStudio tools v3.6 at https://www.omicstudio.cn/tool (accessed on 11 September 2024).

### 4.6. Al^3+^ Tolerance Assay

The effects of different Al^3+^ treatments on plant root growth were measured in peanut seedlings. The seedlings were cultivated in CaCl_2_ solution for 24 h and then transferred to 100 μM AlCl_3_ for 48 h (pH 4.2–4.5). Root growth after Al^3+^ exposure was determined, and the root tips were stained with hematoxylin according to Yao et al. [32]. Fresh root tips (1.0 ± 0.1 mm) were excised, washed in distilled water for 15 min, and immersed in 0.1% hematoxylin (containing 0.01% KIO_3_) for 20 min. Then, the samples were rinsed in running water for a further 10 min, and the root tips were observed with a stereo microscope (SZX7, Olympus, Tokyo, Japan).

Al^3+^ uptake was investigated using inductively coupled plasma optical emission spectrometry (ICP-OES). A total of 1.0 g (Fwt.) of peanut root tips (1.0 ± 0.1 mm) was suspended in 1 mL of 2 M HCl at room temperature for 24 h with occasional shaking and then centrifuged at 12,000× *g* for 30 min. The transparent solution was analyzed by ICP-OES (Varian700, Agilent, Santa Clara, CA, USA).

### 4.7. Photosynthesis and Chl Fluorescence Measurements

A portable GFS-3000 gas exchange and fluorescence system (Heinz Walz GmbH, Effeltrich, Germany) was used for the photosynthetic investigations [33]. Three leaves from each peanut plant and three pots from each treatment were measured. Mature and healthy leaves from the main stem (third open leaf from the top of the main stem to the base of the stem) were used for the analysis. The photosynthetic activity measurements were carried out from 9 a.m. to 12 p.m. on the final day of treatment. The net assimilation rate was measured using a GFS-3000 system (Walz GmbH, Effeltrich, Germany) after the leaves had been dark-adapted for 30 min.

The maximum quantum efficiency of PSII (Fv/Fm) and the maximum rate of electron transfer (ETR_max_) were measured using a PAM-2500 (Walz, Effeltrich, Germany) after 30 min of dark adaptation with dark clips. All measurements were performed between 9:00 a.m. and 12:00 p.m. on the final day of treatment, and the functional leaves on the main stem (third open leaf from the top of the main stem to the base of the stem) were measured.

### 4.8. Transmission Electron Microscopy Observation of Cell Ultrastructure

Tissue samples from the functional leaves and the nearby stems were excised and fixed in 2.5% glutaraldehyde for 12 h at 4 °C. The samples were rinsed twice with 0.1 m phosphate-buffered saline (PBS) for 10 min each time, fixed again in 1% osmium acid for 3 h at 4 °C, and washed twice with PBS as described above. After fixation, the segments were dehydrated in ethanol at graded serial concentrations of 70%, 80%, 90%, and 100%, for 20–30 min at each step. The 100% ethanol step was repeated 2–3 times to ensure complete dehydration. The dehydrated segments were rinsed with 100% acetone and then permeated by graded mixtures of acetone and epoxy resin 618 (Beijing Zhongxing Bairui Technology Co., Ltd., Beijing, China) at ratios of 3:1, 1:1, and 1:3. The segments were polymerized stepwise at 37 °C for 12 h, 45 °C for 12 h, and 60 °C for 48 h. Each segment was sectioned at 70 nm by a Leica UC7 ultra-microtome (Leica, Wetzlar, Germany), double stained using 1% uranyl acetate for 20 min and 1% lead citrate for 7 min at 25 °C, mounted on copper grids, and visualized by a transmission electron microscope (JEOL H7650, Hitachi High-Technologies Corporation, Tokyo, Japan) at an accelerating voltage of 80 kV (magnification is shown in the picture).

### 4.9. Statistical Analyses

IBM SPSS Statistics 23 software (IBM, Armonk, NY, USA) was used for data analysis. The data were presented as the mean ± SD (*n* = 3). Statistical significances were determined using Student’s *t*-test.

## 5. Conclusions

Overall, Al^3+^ stress increases the expression of the gene encoding *AhClpP6*, which is a positive modulator of peanut growth and development. In addition, *AhClpP6* promotes photosynthetic efficiency via enhancing the integrity of chloroplasts, thereby positively contributing to Al^3+^ tolerance in peanut plants. These findings provide a compelling example of how peanut plants enhance the tolerance to exposure to Al^3+^ stress by the ATP-dependent caseinolytic protease system. Moreover, the results reveal a new mechanism of Al^3+^ tolerance which may be used to favor breeding for stress tolerance.

## Data Availability

All data included in this study are available upon request by contacting the corresponding author.

## Supplementary Materials

The following supporting information can be downloaded at: https://www.mdpi.com/article/10.3390/ijms251910416/s1.
