# Supplementary material for: Knockdown of Adenosine 5′-Triphosphate-Dependent Caseinolytic Protease Proteolytic Subunit 6 Enhances Aluminum Tolerance in Peanut Plants (Arachis hypogea L.)
Source: Int J Mol Sci. 2024 Sep 27;25(19):10416. doi: 10.3390/ijms251910416 (PMC11476885; doi:10.3390/ijms251910416)
Supplement: Supplementary file 1 [file ijms-25-10416-s001.zip › ijms-3196107-supplementary.pdf]

Supplementary Table S1. Primer pairs used in this study.

Primers	Sequences (5’-3’)	Purposes
AhClpP6-F	TTAAACGAGCCGGGCCTAAA	Gene clone
AhClpP6-R	AAACTGTGGGCCTCAGAGAAA	
VIGS-*AhClpP6*-F	GTGAGTAAGGTTACCGAATTCATGGTAGCTC CAGCTATATCAGTTCC	VIGS
VIGS-*AhClpP6*-R	GGGACATGCCCGGGCCTCGAGATATTCTGT TTCTAGGACACCATCAATT	
q*AhClpP6*-F	ACAGCCAGTAAATTCGCAAGT	Gene expression analysis
q*AhClpP6*-R	CAATCATAAATTGCCAACACCGAA	
